# Diapause Prevention Effect of *Bombyx mori* by Dimethyl Sulfoxide

**DOI:** 10.1371/journal.pone.0064124

**Published:** 2013-05-13

**Authors:** Takayuki Yamamoto, Keisuke Mase, Hiroshi Sawada

**Affiliations:** 1 Division of Biology, College of Liberal Arts and Sciences, Kitasato University, Sagamihara, Kanagawa, Japan; 2 College of Humanities and Sciences, Nihon University, Setagaya-ku, Tokyo, Japan; Texas A&M University, United States of America

## Abstract

HCl treatment has been, for about 80 years, the primary method for the prevention of entry into embryonic diapauses of *Bombyx mori*. This is because no method is as effective as the HCl treatment. In this study, we discovered that dimethyl sulfoxide (DMSO) prevented entry into the diapause of the silkworm, *Bombyx mori*. The effect of diapause prevention was 78% as a result of treatment with 100% DMSO concentration, and the effect was comparable to that of the HCl treatment. In contrast, in the case of non-diapause eggs, hatchability was decreased by DMSO in a concentration-dependent manner. The effect of DMSO was restricted within 24 hours after oviposition of diapause eggs, and the critical period was slightly shorter than the effective period of the HCl treatment. DMSO analogs, such as dimethyl formamide (DMF) and dimethyl sulfide (DMS), did little preventive effect against the diapause. Furthermore, we also investigated the permeation effects of chemical compounds by DMSO. When treated with an inhibitor of protein kinase CK2 (CK2) dissolved in DMSO, the prevention rate of the diapause was less than 40%. This means that the inhibition effect by the CK2 inhibitor was the inhibition of embryonic development after diapause prevention by DMSO. These data suggest that DMSO has the effects of preventing from entering into the diapause and permeation of chemicals into diapause eggs.

## Introduction

In many insects, development is arrested and diapause is initiated in a specific stage to ensure survival under unfavorable environmental conditions [1]. In the case of the silkworm, *Bombyx mori*, diapause occurs in the late gastrula stage of embryogenesis, when the embryonic cell cycle becomes arrested in the G_2_ phase [2], [3]. The molecular and developmental mechanisms underlying diapause termination and subsequent embryogenesis of *B. mori* remain unexplained. To elucidate the mechanisms, it is essential to delineate embryonic development not only after diapause termination but also before the initiation of the diapause phase [4], [5], [6], [7], [8], [9], [10], [11], [12], [13], [14]. To terminate the diapause, it is necessary to expose the diapause eggs to 5°C for more than 2 months [15]. Alternatively, the HCl treatment is useful to inhibit or interrupt diapause, and the treatment has been used for sericulture in Japan for approximately 80 years. The molecular mechanism of to prevent entry into the diapause by the HCl treatment is not yet understood. However, several hypotheses for the effects of the HCl treatment have been proposed [16], [17], [18], [19].

Dimethyl sulfoxide (DMSO; (CH_3_)_2_SO) is an amphiphilic molecule that is traditionally used as an efficient solvent for a water-insoluble compound, a cryoprotectant of cultured cells [20], [21], a cell fusogen [22], and an enhancer of cell membrane permeability [23]. In addition, DMSO induced differentiation into the human leukemic HL-60 cell [24], [25], and it might induce cardiomyogenesis in P19CL6 embryonal carcinoma cells [26]. The molecular mechanism of this cell differentiation has been reported in several studies [27], [28]. Recently, Wang et al. reported that DMSO extended the lifespan of *Caenorhabditis elegans* [29]. The reports cited above indicate that DMSO has various biological functions. In this study, we show that DMSO has the effects of diapause prevention in *B. mori* and permeation chemicals into eggs. To the best of our knowledge, this is the first report indicating the novel functions of DMSO as a blocker of the entrance of insects diapause and chemical penetration enhancer to deliver an enzyme inhibitor through the chorion into the eggs.

## Materials and Methods

### Insects

The silkworm *B. mori* used in this study was a typical hybrid race (Kinsyu × Syowa). Larvae were reared on Silkmate (Nihon Nosan Kogyo, Ltd., Yokohama, Japan) which was artificial diet for the silkworm. Diapause eggs were obtained from female moths that had been kept under long-day conditions (18L: 6D) at 25°C during embryonic development. Non-diapause eggs were from female moths that had been exposed to 15°C in complete dark as the eggs developed. After copulation, the eggs laid during the first one hour were pooled, sampled at indicated times.

### Chemicals

DMSO, dimethyl formamide (DMF; (CH_3_)_2_NCHO), dimethyl sulfide (DMS; (CH_3_)_2_S), HCl, and beta-carotene were purchased from Wako Pure Chemical Industries, Ltd. (Osaka, Japan), and 4,5,6,7-tetrabromobenzotriazole (TBB) was purchased from Sigma-Aldrich.

### HCl treatment

For artificial diapause termination by HCl, the diapause eggs at 20 hours after oviposition were treated with HCl (specific gravity 1.10) for 60 min at 25°C. After the HCl treatment, eggs were washed in running water and air-dried. The treated diapause eggs had been kept at 25°C until hatched.

## Results

### Diapause prevention effect of *B. mori* by DMSO

To elucidate the preventive effects of DMSO on embryonic diapause, we examined the effects of various concentrations of DMSO, effective treatment times, impact of treatment on the developmental stages, and effects of DMSO analogs.

**Figure 1:**
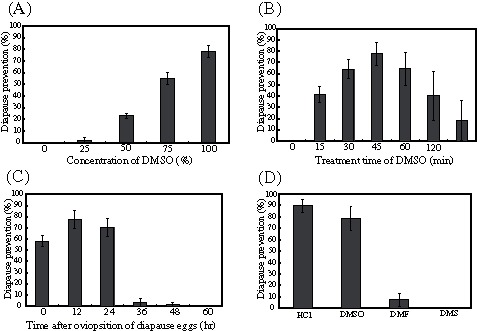
Diapause prevention effect of *B. mori* by DMSO. Effects of various concentrations of DMSO (A). Diapause eggs 12 hours after oviposition treated with 0 to 100% DMSO solutions at 25°C. Each DMSO solution was diluted with deionized distilled water. Effective treatment time of DMSO (B). The diapause eggs 12 hours after oviposition were treated with 100% DMSO for 0 to 120 min. The ∞ indicates unwashed eggs. Effect of the DMSO treatment on the developmental stages (C). The diapause eggs 0 to 60 hours after oviposition were treated with 100% DMSO. Effects of HCl and DMSO analogs (D). HCl treatment as described in Materials and Methods. After treatment, eggs, except for the ∞ of (B), were washed in running water and air-dried at 25°C for 30 min. The treated diapause eggs had been kept at 25°C. The prevention rates of diapause ( =  diapause prevention) were calculated from hatchability within 12 days of treatment with DMSO. The hatchability was calculated using 100 to 150 eggs per one experiment. Each solid bars represent the mean values from five independent experiments with ± S.D. shown by vertical lines.

When using 12-hour-old diapause eggs, the rates of prevention of the diapause increased in a DMSO concentration-dependent manner, as shown in Fig. 1A. With a concentration of 100% DMSO, the rate of prevention was 78%. Treatment times with 100% DMSO varied from 0 to 120 minutes. As shown in Fig. 1B, the most effective treatment time was 45 minutes. In the diapause eggs that were not washed after the DMSO treatment (Fig. 1B, ∞), the hatching rate decreased. We investigated the relationship between developmental stages and the prevention effects by DMSO. As shown in Fig. 1C, DMSO had an effect within 24 hours after oviposition, and the effect decreased significantly after 24 hours. The DMSO analogs were also examined. As shown in Fig. 1D, 100% DMF and DMS had little prevention effect. Furthermore, the prevention rates of diapause after treatment with HCl and DMSO were compared. The prevention rates by HCl and DMSO were approximately 90% and 78%, respectively (Fig. 1D).

### Effect of DMSO on non-diapause eggs

**Figure 2:**
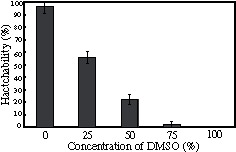
Effect of DMSO on non-diapause eggs. Non-diapause eggs 12 hours after oviposition treated with 0 to 100% DMSO solutions at 25°C. Each DMSO solution was diluted with deionized distilled water. The procedure for the after treatment was the same as that used for the after the DMSO treatment of diapause eggs. The hatchability was calculated using 100 to 150 eggs per one experiment from the number of individuals hatched within 12 days of treatment with DMSO. Each value represents the mean ± S.D. of five independent experiments.

Non-diapause eggs hatch within 2 weeks of oviposition, which differs from diapause eggs. We examined the influence of DMSO on non-diapause eggs. Eggs 12 hours after oviposition were treated for 45 minutes with various concentrations of DMSO, and the hatchability decreased significantly in a DMSO concentration-dependent manner, as shown in Fig. 2.

### Permeation effect of chemicals into diapause eggs by DMSO

**Figure 3:**
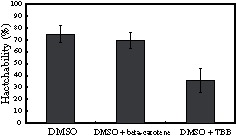
Permeation effect of chemicals into diapause eggs by DMSO. Diapause eggs 12 hours after oviposition were treated with beta-carotene or TBB dissolved in 100% DMSO. The procedure after treatment was the same as that after the DMSO treatment of diapause eggs. The hatchability was calculated using 100 to 150 eggs per one experiment from the number of individuals hatched within 12 days of the treatment. Each value represents the mean ± S.D. of five independent experiments.

To investigate the effect of DMSO on the permeation of chemicals into the eggs, we treated diapause eggs with a beta-carotene or an inhibitor (4,5,6,7-tetrabromobenzotriazole; TBB) of protein kinase CK2 [30], which was dissolved in 100% DMSO. In these experiments, if the chemicals that permeated the eggs affected embryonic development, the hatchability would be changed in comparison with those in a control experiment (Fig. 3, DMSO alone). As shown in Fig. 3, when using 12-hour-old diapause eggs, in treatment with 0.1 mM TBB, hatchability was less than 40%. In contrast, hatchability was unaffected by treatment with 0.1 mM beta-carotene (Fig. 3). The beta-carotene was used as a control experiment for the TBB.

## Discussion

In the present study, we discovered that DMSO prevented entry into the diapause in the silkworm, *Bombyx mori*. The prevention rate of the diapause by the optimal treatment conditions was approximately 78%, and the effect was comparable to that by the HCl treatment. The effect of DMSO was restricted within 24 hours of oviposition of the diapause eggs, and the effective period was slightly shorter than that obtained with the HCl treatment. The prevention effect of the diapause by DMSO was limited to the eggs before the start of pigmentation. The beginning of pigmentation of the diapause eggs by ommochrome, such as a xanthommatin and an ommin [31] signifies the beginning of pigment granules formation in the serosa cells [32]. Therefore, the effective period of DMSO treatment is probably immediately before the pigment granules formation. The serosa cell layer containing the matured pigment granules may be involved in the suppression of the DMSO effect. In contrast, the HCl treatment has the prevention effect even after the formation of pigment granules by an accumulation of ommochrome in the serosa cells. These data may suggest that DMSO and HCl prevent entry into the diapause through different molecular mechanisms.

Most eggs that were unwashed after the DMSO treatment began embryonic development; however, most of them died before hatching. Therefore, the hatching rate decreased. Similarly, when the non-diapause eggs were treated with DMSO, hatchability decreased in a DMSO concentration-dependent manner. In the case of *in vitro* cultured CL1-5 cells (human lung adenocarcinoma cell line), treatment with more than 5% DMSO led to cell death [27]. The report and the above results indicate that excess DMSO has toxicity against cells and DMSO influences the embryonic development after diapause termination and/or non-diapause development.

The degree of DMSO permeation of the eggs cannot be determined because the cells are surrounded by the chorion, which is the outer shell of the egg. HCl is presumed to penetrate through the chorion, including the aeropyle and mycropyle, but the degree of absorption depends upon the structure and thickness of the chorion [33]. The thickness of the chorion differs in each races of the silkworm [33]. Therefore, the optimal treatment condition by DMSO may differ in different silkworm races.

DMSO analogs, such as DMF and DMS, had little preventive effect on the diapause. It is known that DMF and DMS have more toxicity than DMSO. Therefore, DMF and DMS may damage the cells rather than inhibit the diapause. Accordingly, the hatchability within 2 weeks of treatment ( =  diapause prevention rate) was significantly reduced.

When the diapause eggs were treated with TBB dissolved in DMSO, hatchability decreased less than 40%. Phosphorylation by CK2 plays an important role in the embryonic development of the silkworm [10], [14], [34]. DMSO enhances cell membrane permeability [23]. Because of the TBB, which permeated the cells through the chorion with DMSO, CK2 activity was inhibited, and the inhibition may have arrested development.

Human promyelocytic leukemia HL-60 cells are known to differentiate into neutrophils as a result of DMSO treatment [24], [35]. Gailani et al. [36] proposed that DMSO treatment caused rapid down-regulation of *c-myc* mRNA of HL-60 cells and suggested that reduction in *c-myc* expression is necessary for differentiation to occur in HL-60 cells. Kami et al. [26] also reported that Gremlin enhances the determination path to DMSO-induced cardiomyogenesis of P19CL6 cells in a stage-specific manner. These reports reveal an aspect of the molecular mechanism of cultured cell differentiation as a result of DMSO treatment. At present, it is unclear whether the mechanisms of these cultured cell differentiation by DMSO are the same as the mechanism of blocking of the entrance of diapause. Further research will be required to clarify the mechanisms of preventing from entering into diapause by DMSO, and the permeation effect of chemicals by DMSO.
