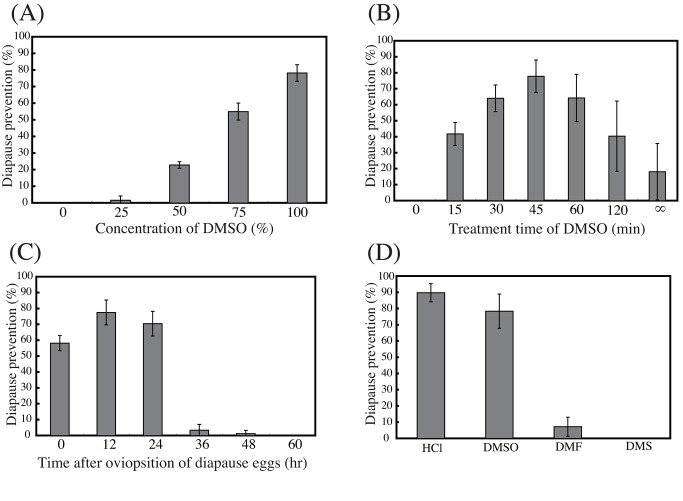# Correction: Diapause Prevention Effect of *Bombyx mori* by Dimethyl Sulfoxide

**DOI:** 10.1371/annotation/b0e62f4f-812f-40b1-aef8-365b229eb2cf

**Published:** 2013-11-08

**Authors:** Takayuki Yamamoto, Keisuke Mase, Hiroshi Sawada

An error was introduced in the preparation of this manuscript for publication. In Figure 1B, the final value on the X-axis is missing. There should be a ∞ under the bar furthest to the right.

You can find the correct figure here: 

**Figure:**